# Effects of rapid weight loss and rapid weight regain on oxidant status and muscle damage in judo athletes

**DOI:** 10.3389/fphys.2026.1953888

**Published:** 2026-09-15

**Authors:** Büşra Özdek, Hamza Budak, Emre Karaduman, Eda Karaduman, Mahmut Esat Uzun, Ali Kerim Yılmaz

**Affiliations:** 1 Faculty of Aksaray University Sports Sciences, Aksaray University, Aksaray, Türkiye; 2 Faculty of Yaşar Doğu Sports Sciences, Ondokuz Mayıs University, Samsun, Türkiye; 3 Faculty of Sports Science, Karabük University, Karabük, Türkiye

**Keywords:** judo, muscle damage, oxidative stress, rapid weight cycling, redox homeostasis

## Abstract

**Background:**

Athletes in weight-class sports frequently use rapid weight loss (RWL) before the official weigh-in to compete in a lower weight category and potentially gain a competitive advantage. After the weigh-in, rapid weight regain (RWG) is commonly applied to restore body mass before competition. However, repeated weight cycling may impair physiological functions, including redox homeostasis and muscle integrity. This study investigated the effects of RWL and subsequent RWG on oxidative stress and muscle damage markers in male judo athletes.

**Methods:**

Eleven male judo athletes (20.8 ± 0.87 years) participated in this repeated-measures study. Assessments were conducted before RWL (pre-RWL), after a 5-day RWL period (post-RWL), and following a 15-h RWG period (post-RWG). Body weight, total oxidant capacity (TOC), total antioxidant capacity (TAC), oxidative stress index (OSI), and myoglobin (Mb) concentrations were measured at each time point.

**Results:**

Body weight decreased by 6% following RWL and remained 4% below baseline after RWG (p<0.001). TAC increased by 20% after RWL and remained 11% above baseline following RWG, whereas TOC and OSI decreased by 12% and 28%, respectively, after RWL before increasing to 33% and 21% above baseline following RWG (p < 0.05). Myoglobin increased markedly by 339% after RWL and remained 42% above baseline following RWG (p<0.05). Furthermore, positive correlations were observed between changes in TAC and Mb during both RWL (r=0.727, p<0.05) and RWG (r=0.609, p<0.05).

**Conclusions:**

RWL and RWG induced distinct changes in redox homeostasis. RWL was associated with an enhanced antioxidant response, whereas RWG increased oxidative stress, indicating that post-weigh-in recovery strategies may substantially influence oxidative balance. These findings emphasize the importance of monitoring both weight-loss and weight-regain phases in weight-class sport athletes.

## 1 Introduction

Weight-class sports are divided into different body mass categories to ensure fair competition by enabling athletes with similar physical characteristics to compete against one another (Artioli et al., 2010). Prior to each tournament, athletes undergo official weigh-in approximately 15–16 hours before competition to determine their respective weight categories (Reale et al., 2017a). Many athletes prefer to compete in lower weight classes under the assumption that facing lighter opponents may confer a performance advantage. Consequently, athletes commonly engage in RWL strategies 1–7 days before competition and subsequently employ RWG strategies after official weigh-ins to regain the lost body mass within a short recovery window (Matthews et al., 2019; Bialowas et al., 2023). RWL and RWG practices have become widely observed across various weight-class sports, particularly in judo, wrestling, and mixed martial arts (Brito et al., 2012; Gann et al., 2015).

Methods used to achieve RWL typically include energy restriction, fluid restriction, dehydration strategies, increased training load, exercise performed in thermal clothing, and sauna use, all of which may impose substantial physiological risks (Lakicevic et al., 2020; Kons et al., 2022). When dehydration is the primary mechanism, these practices have been reported to negatively affect aerobic and anaerobic performance, muscular strength, and endurance, as well as to impair motor skills. Moreover, energy restriction, excessive training loads, and insufficient recovery strategies during RWL may increase physiological stress and induce various metabolic and cellular alterations (Gigou et al., 2010; MacLeod and Sunderland, 2012; Smith et al., 2012; Savoie et al., 2015; Gamage et al., 2016).

One of the key responses to these alterations is oxidative stress. Oxidative stress is defined as a condition resulting from an imbalance between reactive oxygen species (ROS) production and antioxidant defense mechanisms, leading to potential cellular damage in biological systems (Forman and Zhang, 2021). It has been suggested that dehydration, energy restriction, and high-intensity physical loading during RWL may increase ROS production and thereby disrupt redox homeostasis. Indeed, Kowatari et. al., (2001) demonstrated that even body mass reductions below 5% can alter redox balance (Kowatari et al., 2001). Similarly, Nishimaki et al. (2018) reported that a ~5% reduction in body mass increases oxidative stress levels (Nishimaki et al., 2018). Lee et al. (2026) further showed that RWL combined with high-intensity exercise is associated with unfavorable lipid profiles and elevated oxidative stress in athletes (Lee, 2026).

Increased oxidative stress not only disrupts cellular redox homeostasis but may also induce structural and functional alterations in muscle tissue. In recent years, exercise-induced free radical production and oxidative damage have been emphasized as key mechanisms underlying muscle damage (Cheung et al., 2003; Close et al., 2005; Desgorces et al., 2017). Muscle damage is characterized by reductions in muscle strength, limited range of motion, localized swelling, delayed onset muscle soreness, and elevated circulating markers of muscle damage (Mohr et al., 2016).

In this context, Roklicer et al. (2020) reported that judokas who rapidly lost at least 5% of their body mass before competition experienced significant physiological strain and skeletal muscle damage (Roklicer et al., 2020). Coswig et al. (2015) observed significant increases in muscle damage marker levels following RWL in mixed martial arts athletes, while Işık et al. (2018) demonstrated adverse changes in muscle damage markers in wrestlers under-going rapid weight reduction protocols (Coswig et al., 2015; Isik et al., 2018).

When the effects of RWL and subsequent RWG and physiological variables are considered, the literature is limited and does not present a clear consensus. Existing studies have largely focused on the effects of RWL on performance-related variables, whereas the post-weigh-in RWG period has received comparatively limited attention (Kraemer et al., 2001; Kwiatkowska-Pamuła et al., 2017; Barley et al., 2018). However, the RWG period is a physiologically significant process. Furthermore, it has been shown that catabolic hormonal responses induced by caloric restriction and dehydration may persist even after RWG (Cannataro et al., 2020). This suggests that the physiological changes occurring during RWL may not be limited to the weight loss period and may continue during the RWG period. On the other hand, while some studies suggest that RWG may provide athletes with a competitive advantage (Alderman et al., 2004; Reale et al., 2017b), other studies have reported that RWG following RWL has no significant effect on performance outcomes (Daniele et al., 2016; Reale et al., 2017a; Reale et al., 2017b).These conflicting findings indicate that the physiological consequences of rapid weight fluctuations have not yet been fully elucidated and warrant further investigation.

Although a considerable number of studies have examined the effects of RWL on performance, hydration status, and various biochemical parameters, research simultaneously investigating both RWL and post-weigh-in RWG, particularly in relation to oxidative stress and muscle damage markers, remains limited. Accordingly, the aim of the present study was to investigate the effects of RWL prior to competition and subsequent RWG on oxidative stress and muscle damage markers in weight-class athletes. It was hypothesized that both RWL and subsequent RWG would significantly affect oxidative stress and muscle damage markers.

## 2 Materials and methods

### 2.1 Participants

Eleven male judo athletes voluntarily participated in the study. The sample size was determined *a priori* using G*Power software (version 3.1.9.6, Germany). Accordingly, an effect size (f=0.35), a significance level of α=0.05, and a statistical power of 1 − β = 0.80 were specified for a one-group repeated-measures design with three measurement occasions. The expected effect size was based on the observed oxidative stress response reported in a comparable repeated-measures study of judo athletes (Knapik et al., 2019). Under these assumptions, G*Power indicated a minimum required sample size of 10 participants. To account for potential attrition and to enhance the robustness of the analyses, 11 athletes were recruited, and all participants completed the study.

The inclusion criteria were actively practicing judo, having at least 5 years of active training history, previous experience with RWL practices before competition. Exclusion criteria included the presence of any acute or chronic illness, infection, or serious musculoskeletal injury within the previous 6 months, as well as the use of pharmacological drugs (diuretics, laxatives, etc.) or dietary supplements that could affect oxidative stress, muscle metabolism, or hydration status during the study period.

Prior to participation, all athletes provided written informed consent. The study protocol was conducted in accordance with the Declaration of Helsinki and approved by the Ethics Committee of Selçuk University (Approval No: E.1168520).

### 2.2 Study design and measurement protocol

A repeated-measures within-subject design was employed to simulate pre-competition weight management practices commonly observed in weight-category sports. This design was selected specifically to reflect real-world competitive conditions and to allow each athlete to serve as their own control, thereby reducing inter-individual variability in oxidative stress and muscle damage responses.

Previous literature indicates that combat sport athletes typically reduce approximately 5–7% of body mass within 5–7 days prior to competition, followed by a recovery period of approximately 15 hours between official weigh-in and competition onset (Brito et al., 2012; Khodaee et al., 2015; Barley et al., 2019; Matthews et al., 2019).

Based on this evidence, a three-phase protocol was implemented (**Figure 1**):

**Figure 1:**
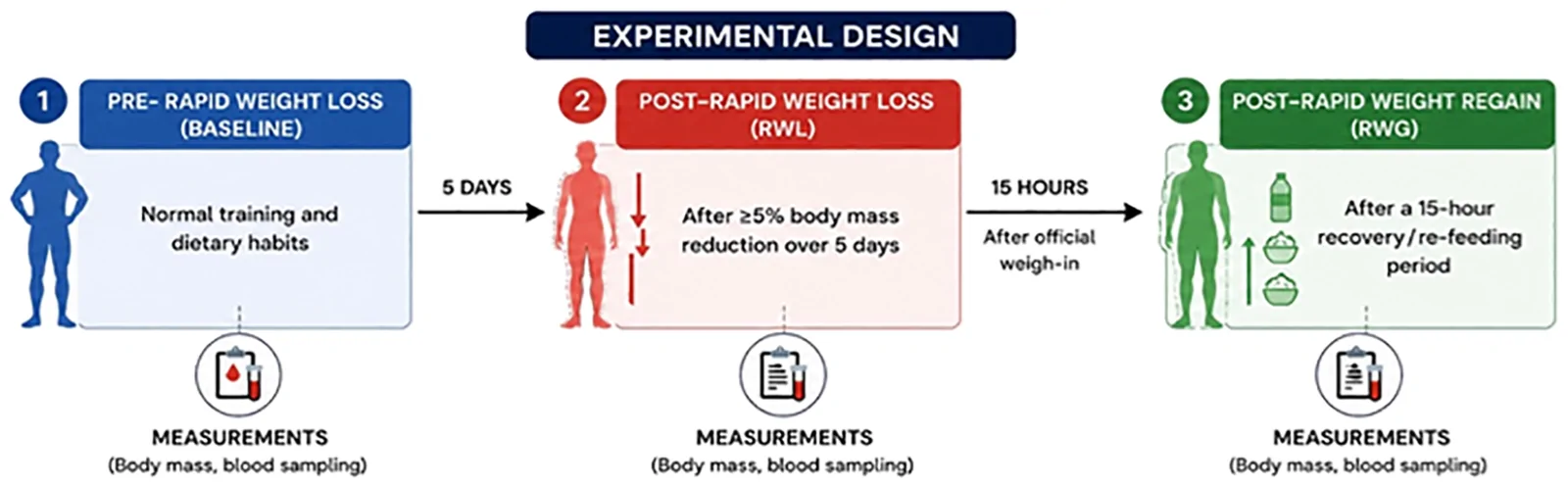
Experimental design.

1. Pre- Rapid Weight Loss (pre-RWL): Normal training and dietary habits.
2. Post–Rapid Weight Loss (post-RWL): After ≥5% body mass reduction over 5 days.
3. Post–Rapid Weight Regain (post-RWG): After a 15-hour recovery/re-feeding period following official weigh-in.

To minimize potential circadian variation, measurements during the pre–RWL and post–RWL phases were conducted at the same time of day. In contrast, post–RWG assessments were scheduled to coincide with the typical competition start time to reflect the athletes’ physiological status at the time of competition. During the study period, participants maintained their habitual training routines. However, during the RWG phase, all structured exercise was prohibited.

### 2.3 RWL and RWG procedures

Athletes were instructed to voluntarily reduce at least 5% of their initial body mass within five consecutive days using their self-selected methods, reflecting real-world practices in combat sports. Allowed strategies included increased physical activity, caloric restriction, fluid restriction, sauna use, and use of sweat-inducing clothing. Participants were instructed to maintain detailed self-reports of their weight-loss strategies; however, no dietary standardization or external intervention was imposed to preserve ecological validity. The use of diuretics, laxatives, and pharmacological weight-loss agents was strictly prohibited. Following official weigh-in, athletes underwent a 15-hour ad libitum recovery period involving self-determined rehydration and feeding practices. No specific nutritional or hydration protocol was imposed during this phase. Body mass was measured at each time point using a calibrated bioelectrical impedance analyzer (Omron Body Composition Monitor KaradaScan HBF-371, Omron Healthcare Co., Kyoto, Japan), under standardized light clothing conditions and without shoes.

### 2.4 Blood sampling and biochemical analysis

Blood samples were collected at each measurement point. The pre-RWL and post-RWL samples were obtained following a 12-h overnight fast and 2 h after the final training session, whereas the post-RWG sample was collected at the end of the 15-h ad libitum refeeding and rehydration period. Samples were drawn into EDTA-containing vacutainer tubes (Becton Dickinson, Plymouth, UK) and immediately centrifuged at 3000 rpm for 10 minutes. Plasma aliquots were separated and stored at −80 °C until biochemical analysis. Plasma myoglobin (Mb) concentrations were determined using an automated immunoassay analyzer (Elecsys 2010, Roche Diagnostics GmbH, Mannheim, Germany) according to manufacturer instructions. TOC and TAC were measured using commercially available colorimetric assay kits (Rel Assay Diagnostics, Türkiye), based on methods described by Erel (2004, 2005) (Erel, 2004; Erel, 2005). TOC was expressed as μmol H_2_O_2_ equivalent/L, and TAC as mmol Trolox equivalent/L. The oxidative stress index (OSI) was calculated using the following formula: OSI = (TOC/TAC).

### 2.5 Statistical analysis

All analyses were conducted using IBM SPSS Statistics (Version 28.0.1.0). All measurements are presented as mean, standard deviation (SD), median, minimum (min), and maximum (max). The normality of the data distribution was assessed using the Shapiro–Wilk test. A one-way repeated measures ANOVA was applied for normally distributed variables (body mass). When significant main effects were detected, pairwise comparisons were conducted using Bonferroni-adjusted *post hoc* tests. Effect size was calculated using partial eta squared (ηp²). For variables that were not normally distributed, the Friedman test was used to examine differences across the three time points. The results of the Friedman test were reported using the chi-square (χ²) statistic. When significant differences were detected, pairwise comparisons were per-formed using Wilcoxon signed-rank tests with Bonferroni correction. Effect size for the Friedman test was reported using Kendall’s W. Spearman’s rank correlation analysis was performed to examine associations between changes in the study variables. Change scores (Δ) were calculated for the pre-RWL to post-RWL, post-RWL to post-RWG, and pre-RWL to post-RWG periods and were subsequently used in the correlation analyses. Percentage change in body weight, oxidative stress biomarkers (TAC, TOC), and myoglobin concentrations was computed using the standard formula: [(ValueB – ValueA)/ValueA] × 100, and these values were subsequently presented in the graphical representations. Statistical significance was accepted at p < 0.05.

## 3 Results

This section presents the results of statistical analyses conducted to evaluate the effects of RWL and RWG on redox homeostasis and muscle damage.

Participant characteristics are presented in **Table 1**. Changes in body weight, oxidative stress parameters, and myoglobin concentrations are presented in **Table 2**. Body weight changed significantly across the experimental protocol (F = 462.680, p<0.001, partial η²=0.979). Pairwise comparisons demonstrated that body weight decreased significantly following post-RWL and increased after post-RWG; however, post-RWG body weight remained significantly lower than pre-RWL (p<0.05).

**Table 1:** Baseline characteristics of the participants.

Variable	Mean ± SD	Med (min to max)
Age (y)	20.82 ± 0.87	21 (20 to 22)
Height (cm)	179.82 ± 4.51	179 (174 to 188)
Weight (kg)	79.05 ± 9.93	77 (66 to 95)
BMI (kg/m^2^)	24.36 ± 2.02	25 (22 to 28)
Sport experience (y)	7.36 ± 0.92	7 (6 to 9)

Data are presented as mean ± SD and Med(min to max). BMI, Body mass index.

**Table 2:** Changes in body weight, oxidative stress markers, and myoglobin concentrations during pre-RWL, post-RWL and post-RWG.

Variable	pre-RWL	post-RWL	post-RWG	p
Body Weight (kg)	79.05 ± 9.93	74.13 ± 9.76^#^*	76.22 ± 10.07*	<0.001
TAC (mmol)	1.49 ± 0.24	1.79 ± 0.29^#^*	1.65 ± 0.24*	0.001
TOC (μmol)	3.53 ± 1.03	3.14 ± 1.58	4.77 ± 2.31^#^*	0.009
OSI (au)	2.37 ± 0.56	1.67 ± 0.58^#^*	2.84 ± 1.18	0.020
Mb (ng/mL)	39.24 ± 21.09	160.28 ± 107.69^#^*	53.22 ± 28.63*	0.001

TAC, Total antioxidant capacity; TOC, Total oxidant capacity; OSI, Oxidative stress index; Mb, myoglobin; *different from pre-RWL; ^#^different from post-RWG.

TAC differed significantly across the three measurement time points (χ²=18.18, p=0.001, Kendall’s W = 0.83). Pairwise comparisons indicated that the TAC was significantly higher at post-RWL than at pre-RWL and post-RWG (p<0.05). Furthermore, the TAC at post-RWG was significantly higher than that at pre-RWL (p<0.05).

A significant difference in TOC was observed across the three measurements (χ²=9.46, p=0.009, Kendall’s W = 0.43). *Post hoc* pairwise comparisons indicated that TOC values at the post-RWG measurement were significantly higher than those at both the pre-RWL and post-RWL measurements (p<0.05). No significant difference was observed between the pre-RWL and post-RWL measurements (p>0.05).

Likewise, OSI differed significantly across the three measurements (χ² = 7.86, p=0.020, Kendall’s W = 0.36). *Post hoc* pairwise comparisons demonstrated that OSI was significantly lower following post-RWL than both pre-RWL and post-RWG (p<0.05), whereas no significant difference was observed between the pre-RWL and post-RWG measurements (p > 0.05).

A significant difference in the myoglobin concentrations was observed across the three measurements (χ²=15.95, p=0.001, Kendall’s W = 0.73). Pairwise comparisons indicated that the myoglobin was significantly higher at the post-RWL measurement than at both the pre-RWL and post-RWG measurements (p<0.05). Furthermore, myoglobin at the post-RWG measurement remained significantly higher than that at the pre-RWL measurement (p<0.05) (**Table 2**).

Furthermore, relative to pre-RWL, body weight decreased by 6% following RWL and remained 4% below baseline following RWG. TAC increased by 20% and 11% at post-RWL and post-RWG, respectively. TOC decreased by 12% following RWL but in-creased by 33% following RWG, while OSI decreased by 28% following RWL and increased by 21% following RWG. Myoglobin increased markedly by 339% following RWL and remained 42% above the pre-RWL value following RWG (**Figure 2**).

**Figure 2:**
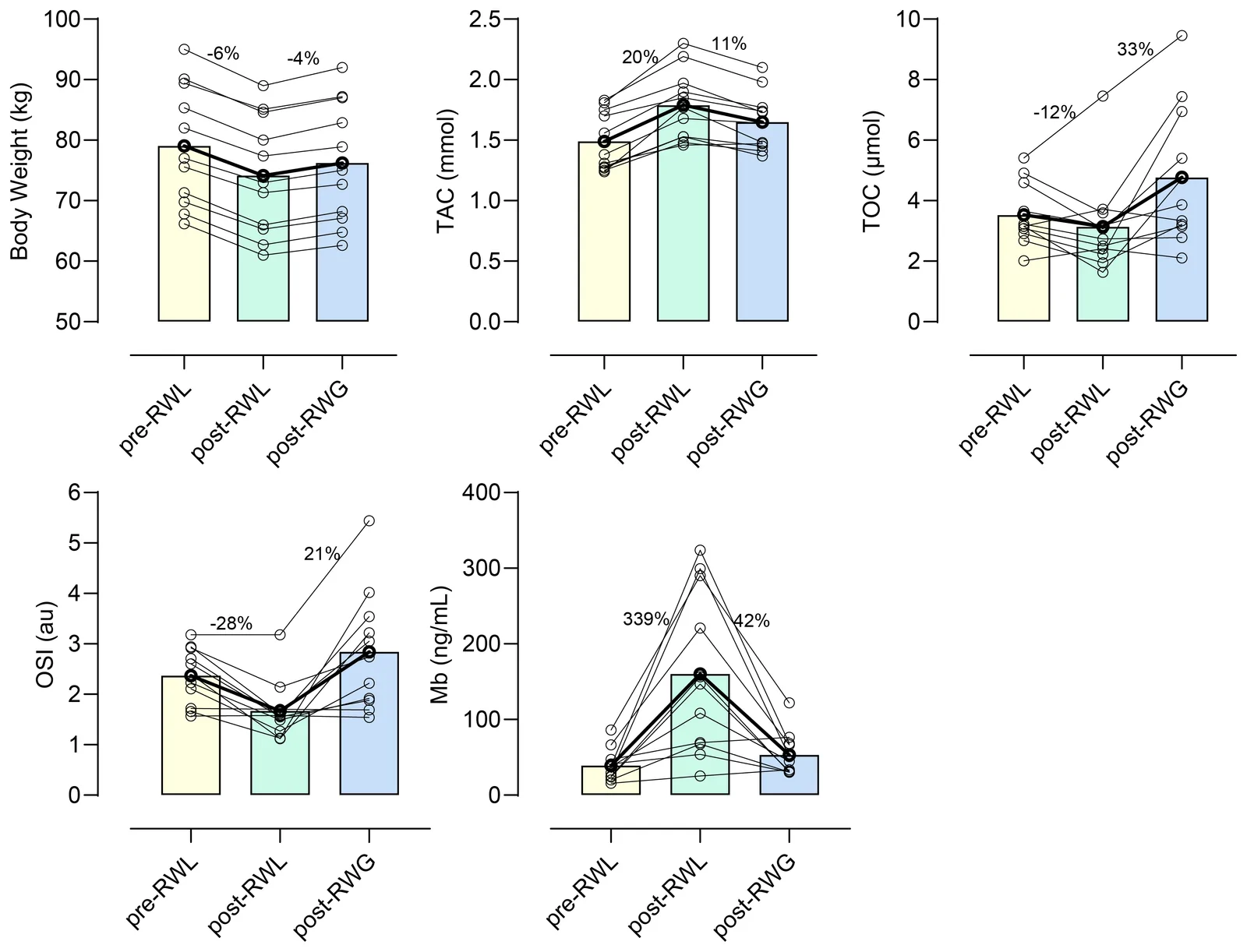
Individual and mean (bold line) changes in body weight, oxidative stress biomarkers (TAC, TOC, and OSI), and myoglobin concentrations during RWL and RWG. Percentage values indicate the relative change from the pre-RWL measurement. TAC, Total antioxidant capacity; TOC, Total oxidant capacity; OSI, Oxidative stress index; Mb, myoglobin.

Correlation analysis of the changes (Δ) following RWL revealed a strong positive correlation between changes in ΔTAC and ΔMb (r=0.727, p<0.05). No significant correlations were found among the remaining variables (p>0.05) (**Table 3**).

**Table 3:** Correlations between changes (Δ) in body weight, oxidative stress, and muscle damage markers post-RWL.

Variables	1	2	3	4
1. ΔBody Weight	—			
2. ΔTOC	-0.400	—		
3. ΔTAC	-0.045	-0.082	—	
4. ΔMb	0.055	-0.318	0.727*	—

Δpost weight loss – pre-weight loss. Values represent Spearman’s rank correlation coefficients (p). *p<.05.

Correlation analysis of the changes following RWG demonstrated a significant positive correlation between ΔTAC and ΔMb (r=0.609, p<0.05). No significant correlations were identified among the remaining variables (p>0.05) (**Table 4**).

**Table 4:** Correlations between changes (Δ) in body weight, oxidative stress, and muscle damage markers post-RWG.

Variables	1	2	3	4
1. ΔBody Weight	—			
2. ΔTOC	-0.136	—		
3. ΔTAC	-0.055	0.100	—	
4. ΔMb	0.318	-0.209	0.609*	—

Δpost weight regain – post weight loss. Values represent Spearman’s rank correlation coefficients (p) *p<.05.

## 4 Discussion

The present study investigated the effects of RWG following RWL on oxidative stress and muscle damage markers in male judo athletes. The principal findings demonstrated that TOC and OSI reached their lowest levels immediately after RWL, decreasing by 12% and 28%, respectively, before increasing to 33% and 21% above baseline following RWG. In contrast, TAC increased by 20% immediately after RWL and remained 11% above baseline following RWG, whereas myoglobin concentrations exhibited the greatest response, increasing by 339% after RWL and remaining 42% above baseline following RWG. These findings suggest that both RWL and the subsequent RWG phase may be associated with phase-specific alterations in redox homeostasis and muscle damage responses in male judo athletes.

Weight loss induced a marked reduction in body weight, which was only partially restored following RWG. Specifically, body weight decreased by approximately 6% following RWL and remained about 4% below baseline despite RWG. Stangar et al. (2022) reported that the mean percentage of pre-competition weight loss among Olympic athletes was 5.8% (Štangar et al., 2022). This value is consistent with previous studies involving judo and other Olympic combat sport athletes, in which RWL ranged from 2% to 10% of body mass (Artioli et al., 2016; Reale et al., 2017b). Similarly, Ceylan, Barley, and Balci (2023) observed significant changes in body mass among judo athletes, reporting a 6.8% reduction before the official weigh-in, followed by a 4.2% increase from the weigh-in to 24 h before competition, resulting in an overall 2.9% difference between the initial and final measurements (Ceylan et al., 2023). Likewise, Dos Santos et al. (2024) reported that RWL is highly prevalent among judo athletes, with competitors commonly reducing approximately 5% of their body mass. Notably, a considerable proportion of athletes reduced more than 5% of their body mass, including 46.2% of male athletes and 27.3% of female athletes (Dos Santos et al., 2024).

This study showed that TAC increased by 20% after RWL and remained at a higher 11% after RWG compared to the pre-RWL period. In contrast to our findings, Knapik et al. (2019) reported no significant changes in TAC before and after RWL (Knapik et al., 2019). Similarly, Reljic et al. (2015) found that the low-calorie dietary intervention implemented during RWL did not induce significant alterations in antioxidant status (Reljic et al., 2015). The authors suggested that the physiological responses observed during RWL were primarily driven by the increased energy expenditure associated with exercise rather than dietary restriction itself, with the oxidative stress response largely resulting from this mechanism. Conversely, regular physical activity is well recognized to enhance TAC (Gomez-Cabrera et al., 2008). Considering the extensive training background of elite judo athletes, the relatively limited alterations in antioxidant status despite exposure to substantial physiological stress may represent an expected adaptive response. Indeed, trained individuals have been shown to maintain a more stable prooxidant–antioxidant balance in response to physiological stressors, including exhaustive exercise (Gomez-Cabrera et al., 2008) and passive or active hyperthermia (Pilch et al., 2014). Several factors may explain the pronounced changes in antioxidant status observed in the present study. First, different biomarkers of the antioxidant system were evaluated compared with those assessed in previous studies, making direct comparisons difficult. Second, the relatively short duration of the weight-loss period and the magnitude of body mass reduction may have elicited more pronounced adaptive responses within the antioxidant defense system. Consistent with this possibility, Knapik et al. (2019) reported that greater weight loss was associated with an increase in various oxidative stress markers. Likewise, Kowatari et al. demonstrated that even body mass reductions of less than 5% may be sufficient to alter redox balance. Another possible contributor to the observed increase in TAC may be uric acid. Uric acid, the end product of purine metabolism in humans, increases significantly in plasma after intense exercise and contributes significantly to TAC (Sautin and Johnson, 2008). Given that athletes are known to prefer strenuous exercise for rapid weight loss, the increase in TAC observed in the present study may be related, at least in part, to the elevated uric acid concentrations induced by the combined effects of intense exercise and RWL. However, since the underlying antioxidant enzymes and uric acid were not evaluated, this interpretation should be confirmed by future studies involving direct measurements of the relevant redox biomarkers.

Furthermore, the present study demonstrated a reduction in TOC following RWL, followed by a substantial increase after RWG. TOC decreased by 12% post-RWL but increased by 33% post-RWG. In the literature, Knapik et al. (2019) reported significant changes in malondialdehyde (MDA) and TOS following RWL (Knapik et al., 2019). Interestingly, the authors observed that both low (<2%) and high (>5%) body mass reductions resulted in smaller increases in MDA, lipid hydroperoxides (LPH), and TOS than moderate weight loss (2–5%). Furthermore, LPH, MDA, and TOS concentrations measured seven days after weight loss were significantly lower than pre-competition values. The authors suggested that this reduction may have been associated with the intense physical exertion performed during competition. Similarly, Pons et al. (2018) demonstrated that six weeks of dietary restriction significantly reduced MDA concentrations in athletes (Pons et al., 2018). In contrast, Suzuki et al. (2003) reported that the combination of intense exercise and energy restriction increased oxidative burst activity in female judo athletes (Suzuki et al., 2003), whereas Finaud et al. (2006) concluded that a 5% reduction in body mass did not increase lipid peroxidation (Finaud et al., 2006). The elevated TOC levels observed in our study following RWG may be attributable to the cumulative effects of the intense exercise per-formed during the RWL period. In support of this interpretation, Nikolaidis et al. (2012) suggested that disturbances in the prooxidant–antioxidant balance may persist for several days after strenuous exercise (Nikolaidis et al., 2012). This prolonged disruption of redox homeostasis may explain why TOC values remained elevated during the RWG phase despite the cessation of exercise. However, it is important to note that direct comparisons among studies should be interpreted with caution, as different oxidative stress biomarkers have been evaluated, each reflecting distinct components and temporal characteristics of the redox response. Furthermore, variations in the magnitude and duration of weight loss, weight-loss strategies, training status, nutritional practices, and the timing of biological sample collection may all contribute to the inconsistent findings reported in the literature.

Oxidative stress index (OSI) values were significantly lower during the post-RWL period than at both pre-RWL and post-RWG. Given that OSI is derived from the TOC/TAC ratio, the reduction in OSI appears to be primarily attributable to the concomitant decrease in TOC and increase in TAC observed following RWL in the present study. Conversely, the increase in TOC together with the decline in TAC following RWG may have contributed to the return of OSI toward baseline levels. Previous studies have generally reported that RWL, particularly when accompanied by severe de-hydration, marked energy restriction, and strenuous exercise, may increase oxidative stress in combat sport athletes (Abedelmalek et al., 2015; Lee, 2026). The discrepancy between our findings and previous reports suggests that the effects of RWL on oxidative stress are not uniform but may depend on several methodological and physiological factors. Differences in the magnitude of weight loss, the methods used to induce weight loss, hydration status, nutritional practices, training background, and individual antioxidant capacity may substantially influence the redox response. Well-trained athletes generally exhibit enhanced endogenous antioxidant defense systems as a result of long-term training adaptations, which may improve the neutralization of exercise-induced ROS. Therefore, it is plausible that the athletes included in the present study possessed sufficient antioxidant capacity to counteract the oxidative challenge induced by the RWL protocol. This interpretation is consistent with the concomitant decrease in TOC and increase in TAC observed following RWL. Similarly, Finaud et al. (2006) suggested that chronic training adaptations may enhance antioxidant defense during the recovery period, thereby contributing to the maintenance of redox homeostasis (Abedelmalek et al., 2015). Consequently, the lower OSI values observed after RWL in the present study may, at least in part, reflect the combined effects of training-induced antioxidant adaptations and the physiological responses elicited during the RWL process. Another important consideration is the timing of blood sampling. Oxidative stress biomarkers exhibit distinct temporal response patterns depending on the characteristics of the exercise performed and the extent of muscle damage. Exercise protocols that induce minimal muscle damage may elicit early but transient changes in redox biomarkers, whereas eccentric or muscle-damaging exercise may result in delayed peaks in oxidative stress markers, occur-ring up to 48–72 h after exercise (Stachteas et al., 2025). Consequently, it is possible that the sampling schedule employed in the present study did not coincide with the peak oxidative stress response, which may partly explain the lower OSI values observed following RWL.

Myoglobin (Mb) concentrations increased by 339% following RWL. Although Mb concentrations decreased after RWG, they remained 42% above pre-RWL levels. Similarly, Roklicer et al. reported increases in muscle damage biomarkers, including creatine kinase (CK) and aldolase (ALD), in 18 male judo athletes undergoing RWL, suggesting that these increases were primarily associated with the greater training load during the weight-loss period. Likewise, Işık et al. (2018) demonstrated substantial muscle damage following an approximately 5% reduction in body mass achieved through fluid and food restriction, intensive exercise, and sauna use in wrestlers (Isik et al., 2018). Likewise, Özkan & İbrahim (2016) reported significant increases in CK concentrations among athletes who lost more than 5% of their body mass through dehydration (Ozkan and Ibrahim, 2016). In addition, Drid et al. (2019) investigated the effects of an approximately 5% body mass reduction induced by caloric restriction in male judo athletes and demonstrated significant increases in serum creatinine concentrations (Drid et al., 2019). Collectively, these findings indicate that RWL induced through different strategies, including dehydration and dietary restriction, may influence biomarkers of muscle damage. Overall, the present findings suggest that RWL practices in elite judo athletes are associated with alterations in myoglobin concentrations. Although RWL is commonly perceived as a strategy to enhance competitive performance, these practices may induce skeletal muscle damage, potentially compromising athletes’ physical performance profiles (Trivic et al., 2020).

The present study demonstrated that significant positive correlations were observed between changes in ΔTAC and ΔMb during both the post-RWL and post-RWG phases. Whereas increased myoglobin concentrations reflect disruption of muscle membrane integrity and muscle damage, the concomitant increase in TAC may represent a compensatory antioxidant response to the elevated physiological stress. In other words, the activation of antioxidant defense mechanisms may occur in parallel with the increase in muscle damage. This response may reflect the redox adaptations developed through long-term training in elite athletes. Indeed, high-intensity anaerobic exercise is known to induce oxidative modifications in both skeletal muscle and the systemic circulation. Furthermore, lactate accumulation, metabolic acidosis, catecholamine release, and the post-exercise inflammatory response associated with supramaximal exercise have all been reported to contribute to the generation of ROS (Groussard et al., 2003). Considering that athletes generally maintain high-intensity training throughout the RWL period, it is plausible that the associations observed between muscle damage and redox biomarkers in the present study are related to these physiological mechanisms.

Nevertheless, chronic anaerobic training has also been shown to induce protective adaptations against oxidative stress. Regular exposure to high-intensity training may enhance antioxidant enzyme activities, thereby limiting exercise-induced oxidative damage (Bloomer and Goldfarb, 2004). As the athletes included in the present study were elite judo competitors with many years of national and international competitive experience, the absence of a marked increase in the oxidative stress index despite elevated myoglobin concentrations may be explained by enhanced endogenous antioxidant adaptations. This interpretation is also consistent with the positive association observed between ΔTAC and ΔMb.

Finally, it is well established that athletes commonly restrict fluid intake during RWL (Ceylan et al., 2022). Consequently, reductions in plasma volume and the development of hemoconcentration should be taken into consideration. Therefore, it is possible that part of the observed changes in myoglobin and oxidative stress biomarkers may reflect hemoconcentration rather than exclusively representing true physiological responses.

The findings of the present study have several practical implications for weight management in weight-category sports. The phase-specific changes observed in oxidative stress and muscle damage biomarkers suggest that rapid weight cycling should be carefully managed in weight-category sports. Coaches and nutritionists should work collaboratively with athletes to develop individualized weight-management strategies that avoid unnecessary or excessive RWL and allow sufficient time for recovery before and after competition. The post-weigh-in RWG period should also be considered an important component of weight management rather than being viewed solely as a strategy for restoring body mass before competition. Regular monitoring of athletes during both RWL and RWG may help identify unfavorable physiological responses associated with repeated weight cycling.

Several limitations of the present study should be acknowledged. First, the relatively small sample size and limited range of biochemical biomarkers may limit the generalizability of the findings. Future research should be expanded larger sample groups and larger biochemical parameters to understand the changes occurring in the RWL and RWG periods. Second, hydration status and detailed dietary intake were not directly assessed, and the homogeneity of weight-loss strategies could not be established. These limitations restricted our ability to comprehensively assess their potential contributions to the observed changes in oxidative stress biomarkers. Future studies should include detailed assessments of hydration status, dietary intake, and weight-loss strategies. Another limitation of this study is the lack of monitoring of training load and direct assessment of plasma volume. Future studies examining these parameters could provide a more comprehensive understanding of the physiological responses associated with rapid weight cycling.

## 5 Conclusions

RWL significantly reduced body weight, and RWG partially restored it without reaching baseline values. Significant changes were observed in oxidative stress and muscle damage markers throughout the protocol. TAC and myoglobin increased following RWL, whereas TOC increased after RWG. In contrast, the oxidative stress index decreased after RWL and returned to baseline levels following RWG. Correlation analyses showed no significant relationship between changes in body weight and changes in oxidative stress or muscle damage markers during either phase. However, changes in TAC were positively correlated with changes in myoglobin in both the RWL and RWG periods. Overall, rapid weight cycling in elite judokas related significant alterations in oxidative stress and muscle damage responses.

## Data Availability

Requests to access these datasets should be directed to BÖ, busraozdek95@gmail.com.
